# Faecal Pathogen Flows and Their Public Health Risks in Urban Environments: A Proposed Approach to Inform Sanitation Planning

**DOI:** 10.3390/ijerph15020181

**Published:** 2018-01-23

**Authors:** Freya Mills, Juliet Willetts, Susan Petterson, Cynthia Mitchell, Guy Norman

**Affiliations:** 1 Institute for Sustainable Futures, University of Technology Sydney, Level 10, UTS Building 10, 235 Jones Street, Ultimo, NSW 2007, Australia; juliet.willetts@uts.edu.au (J.W.); Cynthia.Mitchell@uts.edu.au (C.M.); 2 Water & Health Pty Ltd., P.O. Box 648, Salamander Bay, NSW 2317, Australia; s.petterson@waterandhealth.com.au; 3 School of Medicine, Griffith University, Parklands Drive, Southport, QLD 4222, Australia; 4 Water and Sanitation for the Urban Poor, 10 Queen Street Place, London EC4R 1BE, UK; gnorman@wsup.com

**Keywords:** pathogens, urban sanitation, wastewater, public health, risk assessment, decision making, faecal waste, options assessment

## Abstract

Public health benefits are often a key political driver of urban sanitation investment in developing countries, however, pathogen flows are rarely taken systematically into account in sanitation investment choices. While several tools and approaches on sanitation and health risks have recently been developed, this research identified gaps in their ability to predict faecal pathogen flows, to relate exposure risks to the existing sanitation services, and to compare expected impacts of improvements. This paper outlines a conceptual approach that links faecal waste discharge patterns with potential pathogen exposure pathways to quantitatively compare urban sanitation improvement options. An illustrative application of the approach is presented, using a spreadsheet-based model to compare the relative effect on disability-adjusted life years of six sanitation improvement options for a hypothetical urban situation. The approach includes consideration of the persistence or removal of different pathogen classes in different environments; recognition of multiple interconnected sludge and effluent pathways, and of multiple potential sites for exposure; and use of quantitative microbial risk assessment to support prediction of relative health risks for each option. This research provides a step forward in applying current knowledge to better consider public health, alongside environmental and other objectives, in urban sanitation decision making. Further empirical research in specific locations is now required to refine the approach and address data gaps.

## 1. Introduction

There is increasing recognition that decisions about sanitation infrastructure for developing country cities should be based on choosing investments that will deliver the greatest benefits to society, however, there are limited tools, data and approaches to accomplish this. Investment in latrine subsidies, faecal sludge management, or wastewater treatment facilities by government or international agencies is often done without an understanding of where faecal pathogens are released within a city, how people are exposed, or the potential effectiveness of investments to reduce disease. While protecting health is a frequently espoused initial driver for sanitation improvements, it is usually not explicitly considered when selecting sanitation improvement options or treatment targets. Current decisions are generally based on capital cost or assumed benefits of individual technologies or practices [1], rather than an understanding of where in a given urban context the most significant public health risks lie, and which improvement options will best address these [2]. Engineers have traditionally focused on environmental objectives, with treatment primarily designed to protect downstream waterways, rather than to address faecal pathogen pathways in the urban environment. In addition, as observed by Mitchell [3], there has been limited discussion of pathogens, other than in intentional sludge or wastewater reuse. It is not surprising then, that authors have recently noted that some sanitation interventions may not do enough to mitigate possible routes of transmission [4]. 

This paper sets out to tackle this issue, by developing a source-pathway-receptor conceptual approach which we then use as the basis for a preliminary model to compare health risks of sanitation options in urban environments. Sanitation decision-making must consider diverse aspects, such as environmental impacts, financial costs, resource recovery and reuse, etc.; but this paper is limited to consideration of public health impacts, because although the health significance and pathway complexity of these impacts are recognised [5,6,7,8], they typically receive scant consideration in actual decision-making about sanitation infrastructure and services. 

Linking sanitation services and health impacts is complex. Whilst recent epidemiological studies have developed estimates of the health impacts associated with inadequate sanitation, including an annual 280,443 diarrhoeal deaths in low and middle-income countries [6], an estimated 5 million disability-adjusted life years (DALYs) lost globally due to soil transmitted helminth infections [9], and inadequate sanitation associated with trachoma, schistosomiasis and stunting [4]. In contrast, there have been mixed results from incidence-based and cross-sectional studies measuring the health impact of sanitation. Two studies in Brazil found 22–60% reduction in child diarrheal disease associated with sewerage [10,11], however other studies found minimal positive health impacts from sanitation improvements [12,13,14]. These studies highlight the difficulty in measuring and comparing health impacts due to improved sanitation [15]. It is unlikely that the requisite studies to conclusively prove the health impact for the breadth of possible urban sanitation improvement options would be feasible or relevant [16]. However, while the magnitude of direct health impacts is uncertain, it is generally accepted that inadequate sanitation and exposure to faecal pathogens affect health, and there is growing evidence that the burden of disease may be higher due to long term effects of environmental enteropathy and stunting [17,18].

A particular challenge in the prediction of health risks from sanitation improvements is the complex and interconnected pathways from excreta to human exposure in poor-sanitation urban environments. While investment in urban sanitation has traditionally focused on centralised wastewater treatment plants at the city’s periphery, it is now evident that a significant proportion of faecal waste does not reach treatment and is released to the environment at various points along the sanitation service chain (containment, conveyance, treatment and disposal from defecation to disposal) [8]. Further adding to the complexity of urban sanitation is the large range of pathogens and numerous transmission pathways: direct and indirect water ingestion; transmission to skin through soil or water; ingestion via hands, food and fomite; vectors proliferating in water; and contaminated aerosols [6]. The consequences of this interconnectivity are highlighted by the findings that health benefits may be more influenced by achieving a threshold level of community coverage rather than an individual’s access to an improved toilet [17]. Without understanding and considering how sanitation can interrupt one or more of these pathways, it can be difficult to interpret the effect of sanitation interventions on health [14].

Recognition of these issues is growing, with the emergence of new tools and approaches that identify the inadequacies of current urban sanitation management and the need for a more holistic approach that considers the entire sanitation service chain. The key literature source on pathogen removal, Feacham [19], is currently being updated by the Global Water Pathogen Project (GWPP), in which a global panel of experts are synthesising available research on water pathogens and their removal in sanitation systems, shared in an online platform (GWPP-2007). Pathogen exposure and health risk assessment are increasingly being applied to urban sanitation [5,6,7,20,21,22] which is often based on detailed field work in selected locations. These studies have highlighted challenges in monitoring pathogens in developing countries where analyses are limited due to cost and ability to measure all types of pathogens and various pathways, and results can be inconclusive.

Despite these emerging tools, assessments and data, there is not yet a systematic approach to prioritise and compare alternative sanitation improvements in terms of likely health risks and hence the approach proposed in this paper. The authors recognise that while the focus of this paper is on protecting public health, other important aspects (such as costs, environment risks, water and nutrient reuse) are critical to sound urban sanitation decision making, and we would envisage the approach presented to be used in combination with other types of analyses. 

## 2. Methods 

### 2.1. Literature Review

The desktop literature review was focused on a gap analysis covering the following areas of enquiry: evidence of consideration of health risks in sanitation assessment tools or planning approaches; critical pathogen release and exposure pathways associated with urban sanitation, and potential for defensible generalisations; available data on pathogen log_10_ reduction in sanitation service chain component systems and reported pathogen concentrations at key points of exposure relevant to developing country cities. 

Journal papers and reports were sourced through targeted searches of academic literature and published reports from the last 10 years to July 2017, using keywords “sanitation” or “wastewater” and the following terms: tools, planning, options, decision, flow analysis, pathogen, health risk, health impact, exposure and risk assessment. The search was not geographically restricted however papers were assessed for relevance to developing country context. In addition, the search was limited to English language. Keyword based searches were conducted in the Web of Science, Scopus and Google Scholar databases; as well as relevant global conference proceedings. We also drew on advice from a range of experts in this field (see Acknowledgements) to identify key literature and provide additional background on the intended scope, limitations and potential to expand existing urban sanitation tools. 

### 2.2. Develop and Test a Conceptual Approach 

We prioritised extension of existing tools in developing our conceptual approach and model. However, a key difference in our intent and perspective was a focus on explicitly looking to compare different possible sanitation improvement options. We adopted a source-pathway-receptor concept and quantitative microbial risk assessment (QMRA) to enable a holistic approach to examine the full pathway including excretion of faecal matter, its fate in an urban environment, and exposure of children and adults and the resultant potential for illness and disease. This paper considers hazard to be the presence of pathogens in the public environment and health risk to be the probability of infection and consequent health outcomes, determined through an understanding of hazard and exposure. The conceptual approach was then tested through a spreadsheet-based model and applied to a hypothetical case (based on key aspects of the situation of Dhaka in Bangladesh) to illustrate the application and its use in option comparison. Inputs and assumptions in the model were based on available literature and are discussed further in the Results section and provided in the Supplementary Materials. The intent was to broadly test the source-pathway-receptor concept in practice, including consideration of the impacts of chosen assumptions, sense-checking outputs with values reported in the literature, and illustrating the types of outputs and insights this approach could potentially offer. An international reference group (see Acknowledgements) including health and sanitation professionals critiqued the model during its refinement. 

### 2.3. Limitations

The development of the conceptual approach and model were necessarily limited by data availability, particularly with regards to predicted system log_10_ reductions and reference pathogen concentrations in the environment for all pathogen types considered. The detailed research synthesis of the GWPP is expected to provide data to inform many parts of this work, but was not complete at the time of writing; therefore values used were based on available literature. Similarly, for the illustrative case study, data on prevalence of disease and expected exposure behaviours were not based on detailed empirical research on Dhaka’s health status or exposures, but rather on selected literature. The scope was also delimited to sanitation improvements for the general public rather than farmers or sanitation workers, for which significant and valuable other work on reuse and risk reduction exists [23,24]. Further aspects of the data limitations and the advantages and disadvantages of using QMRA are described in the Section 4. 

## 3. Results

This section comprises three components. Firstly, the scope of existing tools and approaches to inform decision-making from a public health perspective is assessed. Secondly, the new conceptual approach is presented including the theoretical basis of the proposed components and calculations. Finally, we present illustrative findings of its application in a hypothetical case study.

### 3.1. Assessment of Existing Tools

Various tools have been developed to inform urban sanitation, including assessments of service status, risk assessment approaches and tools to support financial and technical and planning. We assessed recent tools for their existing or potential contribution to inform decision-making from a health perspective, with results summarised in Table 1. Many of the limitations described are not a direct critique of the tools themselves, but rather arise from differences between their intended purpose and our framing.

Our review found that while several tools consider health risks in urban sanitation, as currently conceived, none were found to explicitly link an existing sanitation situation with health risks in a way that could directly inform sanitation planning. In particular, gaps were identified as regards systematic assessment of the source(s) of pathogens entering in the environment, the relative significance of the different faecal waste discharges to the environment, and the influence of variations in removal of different pathogen classes along the sanitation service chain and in urban environments. 

### 3.2. Conceptual Approach to Link Pathogen Flows with Health Risks to Support Decisions

**Figure 1:**
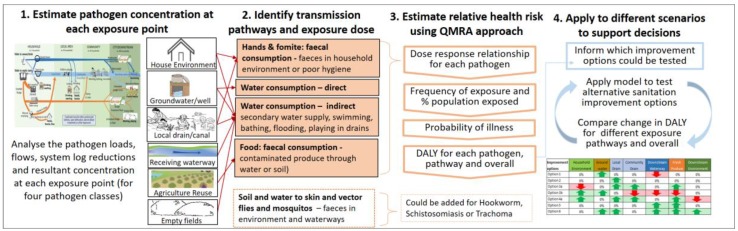
Conceptual approach to compare health risks of urban sanitation improvement options.

In this section, we present and justify the proposed conceptual approach to link pathogen concentrations with health risks in the context of urban sanitation decision-making. Recognising that not all discharges of faecal waste to the environment are of equal concentration, nor are all exposure pathways of equal consequences, a source-pathway-receptor approach was used. This provides a pragmatic yet robust basis to simplify the complexity of an urban sanitation system and is often applied in groundwater studies to assess and mitigate environmental pollution risks [47]. The conceptual approach comprises four interlinked components (see Figure 1): (i) analysis of the “sources” of faecal matter—considering all forms of sanitation and the fluxes of pathogen load across the service chain’s various “pathways”—and resultant pathogen concentration at each exposure point; (ii) identification of relevant transmission pathways and related exposure behaviours; (iii) linkage of pathogen exposure to relative predicted health consequences in “receptors” (both adults and children); (iv) an iterative process of selecting alternative sanitation improvement options (based on the location of identified health risks) and comparing predicted effects of these options.

#### 3.2.1. Estimation of Pathogen Flows and Concentration at Each Exposure Point

**Figure 2:**
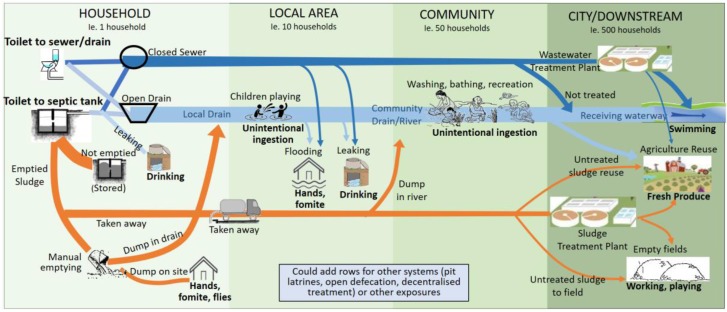
Example system diagram of flow divisions and related exposure points.

The pathogen concentration at each point of exposure is an important variable in the quantitative estimation of health risks from pathogens in the environment. The proposed approach extends the shit-flow diagram (SFD) approach [25]. While the SFD estimates the division of faecal waste flows based on population, our approach considers wastewater flows and pathogen loads as well as their removal, reduction and dilution across the service chain to estimate pathogen concentrations at identified potential points of exposure. The SFD provides a systematic method to assess the faecal waste discharge to the environment from multiple technologies across the sanitation service chain, and the accompanying SFD report often includes further details on the locations of discharges [26] and therefore provides a useful starting point. Building from this, the city’s sanitation system is analysed to understand the flow divisions and interlinkages of multiple flow pathways and the related potential exposure points in an urban context (see Figure 2 for a typical example). Such a diagram could be developed using a participatory approach, as detailed in the Sanitation Safety Planning (SSP) Modules 2 and 3 [24]. It is essential to clearly define the system boundary and scale being considered to set the boundaries of the assessment, while also considering the different scales within the system where faecal waste is discharged or exposure occurs, such as the household, local, community and wider/city scales. Input water volumes must also be estimated. These are likely to differ according to local conditions, such as water availability and cultural practices, household infrastructure (such as whether both blackwater and greywater is flushed with faeces), and local infrastructure (for example, whether wastewater flows are combined with stormwater or other flows). 

Our approach considers input pathogen load in relation to an assumed level of infection prevalence, and distinguishes different pathogen types. The prevalence of infection and hence excreted pathogen loads vary regionally, seasonally and with age [48]. Prevalence can be an important parameter in assessing effectiveness of sanitation infrastructure; most notably the impact of sanitation improvement is expected to be higher in areas with higher initial incidence of diarrheal disease, as confirmed by empirical studies [10]. Input pathogen load can be estimated based on literature values for shedding density for each reference pathogen for an assumed level of infection prevalence, based on local data or relevant estimates. Our approach distinguishes the four pathogen classes (bacteria, protozoa, virus, helminths), rather than relying on *E. coli* alone as an indicator organism. The reasons for this include the demonstrated differential behaviour and response of different pathogen classes in treatment [49,50], the weak relationship between the concentration of *E. coli* and other human pathogens [51], and the differential infective doses, ability to induce human immunity and latency periods of different pathogens [52]. 

Finally, an important aspect of the proposed approach is the application of log_10_ reduction factors to simulate the removal, inactivation or dilution of pathogens in various “sub-systems” (septic tank, filtration through soil, conveyance along drains, waterways, etc.). This aspect of the approach presents perhaps the greatest challenge in assigning meaningful generic assumptions. Existing literature demonstrates extensive variation in log_10_ reductions between and within different pathogen groups in formal treatment units, and particularly in the environment [22]. Equally, there is a lack of knowledge and therefore debate on appropriate assumptions concerning how different pathogen types segregate into liquid (effluent) and solid (sludge, sediment) phases (Kolsky and Evans, pers. comm.). Regardless, it is possible to make estimations and assumptions (and test the sensitivity of these) based on existing literature, as described further in Section 3.3.

#### 3.2.2. Identification of Transmission Pathways and Exposure Dose

Exposure to faecal pathogens in urban areas and elsewhere can occur through multiple transmission pathways: faecal-oral (direct or indirect water consumption, food, fomite, fingers), dermal contact, vector or aerosol [24], with the predominant transmission pathway depending on the pathogen, local infrastructure and human behaviour [53]. In a contaminated urban environment, there are numerous and complex exposure pathways. Hence reducing one transmission pathway alone may not be sufficient to reduce disease incidence [7]. Instead, considering the multiple predominant exposure pathways for this transmission is essential to design and implement systems that lead to decreased risk of disease [22].

Recent studies assessing the health risk related to urban sanitation have identified a number of dominant exposure pathways, with findings influenced by the study scope and site of assessment. Examples include: ingestion of fresh produce [7]; swimming, playing or bathing in open drainage channels [5,34]; ingestion by urban farmers [21]; and ingestion from drainage canals and produce [35]. Based on this evidence, our conceptual approach adopted six generic exposure points, noting that these could and should be locally discussed and determined. The six exposure points are: (i) household environment (hands, fomite); (ii) groundwater (drinking water); (iii) local drain (flooding, children playing); (iv) community drains and downstream waterways (washing, bathing, swimming); (v) agricultural reuse (fresh produce); and (vi) farm land (accidental ingestion). While vector, skin and aerosol exposure pathways are also important, there are limited quantitative approaches to estimate the health risk from pathogens in the environment [54], and these pathways therefore have not been considered at this stage. Adult and child exposures were considered separately, as the transmission pathways and exposures can vary significantly in low-income urban environments [18]. 

In this study, the pathogen concentration at each exposure point was calculated as the sum of the pathogen load divided by flows discharged to that exposure point from the different pathways. This assumes that individuals across the study area are exposed to the same concentration, independent of their individual sanitation practice. A more finite or spatial approach could better consider the influence of local sanitation types on pathogen concentration. Equally important to exposure points are the factors that influence exposure risk: exposure quantity, frequency and population exposed. These values are likely to be context-dependent: this study drew on data from a number of recent risk assessments that involved household and field surveys [5,7,21,33]. Applying our proposed approach in a specific location could involve use of existing methods to consider local exposure pathways [SSP module 2, 24] or direct data collection on local exposure behaviours from household surveys, focus group discussions and transect surveys [7,20].

#### 3.2.3. Linking Exposure with Health Risk

Since the overall objective of this research was to compare sanitation improvement options (not to accurately quantify predicted health impacts), in this paper “health risk” is understood to be a relative risk for the assessed sanitation options. There are many factors that influence the pathogen-host relationship [22] including factors related to pathogens themselves (e.g., residual load due to latency, persistence/die-off, infective dose, species/strain) and factors relating to the host (e.g., immunity, whether natural or acquired through infection or vaccination; age and sex; health status; nutrition/diet; hygiene; season). In the context of comparing sanitation options, many of these factors remain consistent across the alternative sanitation options, since the pathogen context and the receptor population are the same. This reduces the relative complexity and may reduce the impact of potential errors in data-constrained situations [55].

A range of possible approaches to develop links to health risk were considered, and QMRA was selected as the most appropriate and useful. QMRA is supported by World Health Organization (WHO) and provides a systematic way to use scientific information to quantitatively inform and compare risk management options, with more sensitivity to compare inventions than epidemiological studies and more precise than qualitative or semi-quantitative risk assessments [56]. It is commonly used to estimate risks to human health by predicting infection or illness rates given concentrations of pathogen, exposure assessment, health effects assessment and risk characterization [56]. 

QMRA has been applied to urban sanitation in developing countries to determine the magnitude of risk [5,21,33,34,35] and the potential log_10_ reduction achievable by different treatment or interventions [57]. While these studies bear similarities to the approach suggested in this paper, and demonstrate that QMRA can be a useful and important tool for use in urban sanitation, they do not carry the same aim of comparing a range of alternative sanitation options on the basis of relative health risk considering the local context. Of those that assessed sanitation improvements, Labite [5] compared interventions based on expert opinion of the avoided DALY and Surinkul [31] only considered exposure in the context of reuse, while other papers did not quantitively assess sanitation improvement options. Our conceptual approach applies the standard QMRA methodology [56,58], using dose-response models [58] and DALY/case values from literature [59]. DALY per person and per population was the health outcome chosen to compare options, as this allows for the comparison of health risk based on multiple pathogens [59]. Use of QMRA certainly has limitations, and these are considered in the Section 4.

#### 3.2.4. Application to Different Scenarios to Support Decisions

The final component of the proposed approach includes two interlinked steps of: (i) development of possible alternative sanitation improvement options, and (ii) comparison of the related predicted health risks (in DALY) for different exposure pathways. Analysis of the existing sanitation situation through the previous steps provides insight into those pathogens and pathways that contribute the most to health risk, thereby pointing to possible solutions that could best address those pathogens and pathways. A range of possible options, may then be analysed by changing input data, and the relative health risk for each exposure may be compared with the base case. Sensitivity analysis on a range of parameters assists in determining the assumptions driving the model, and testing outputs against published literature values or local data can assist in their validation. It is envisaged comparison of options on the basis of the relative health risk could then be integrated into a broader decision-making framework that includes relevant costs for sanitation infrastructure investments and also considers environmental or other objectives.

### 3.3. Illustrative Application

**Figure 3:**
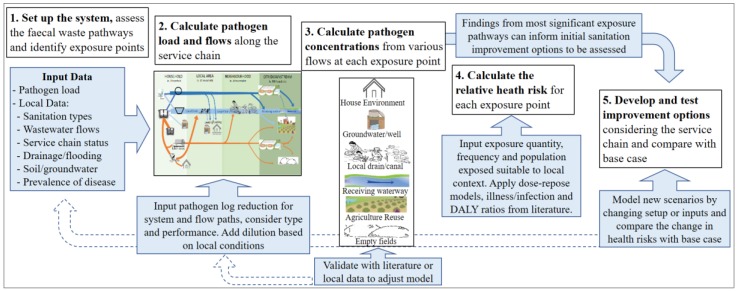
Steps of the spreadsheet-based model developed to apply the conceptual framework.

To test the conceptual logic and the extent to which the approach could be applied based on current knowledge, data and tools, a spreadsheet-based model was developed. This section details the model and its application to a hypothetical case, using data from Dhaka (Bangladesh) [60] based on literature only and no field research or local consultation. As additional assumptions were required for the purpose of modelling, all results presented here are solely for illustration of the modelling process and model outputs, and should not be taken as real results for Dhaka. We present the modelling steps (see Figure 3) in the context of the hypothetical case, including the status of existing evidence and relative reliability of assumptions in each part of the model. The model is at a preliminary stage, suitable for the purposes of demonstrating and illustrating the conceptual approach only. Further development is required to test inputs and assumptions of particular urban contexts, including validation of the inputs and sensitivity testing. This paper therefore only presents an illustrative example rather than quantitative results. The intent is to demonstrate the types of model outputs and therefore show how the conceptual approach could feasibly support decision-making. 

#### Description of Illustrative Case

The illustrative case was primarily based on data from the Dhaka Bangladesh SFD report [60] shown in Table 2 with additional parameters added for the purposes of the modelling exercise. This case represents a typical situation in which significant proportions of faecal matter are discharged to the urban environment, at each of the containment, conveyance and disposal steps in the service chain. 

*Step 1. Set up the system*: The scale of this assessment was set at the city level as per the SFD, noting that smaller community/sub-sections such as an urban slum could also be modelled where more appropriate. The SFD approach was used as a basis to estimate the division of faecal waste flows for each type of sanitation in the service chain, the types of conveyance (e.g., vacuum tanker), the location of sludge discharge or dumping (e.g., next to the pit, in the local drain, to downstream waterway or land), and the destinations of flows not reaching the treatment system (i.e., leaking, flooding, diverted to river). This example was limited to water-based sanitation including toilet-to-drain, toilet-to-sewer and toilet-to-septic-tank, although other sanitation systems and scales are feasible. Pathogen inputs were estimated from available literature. A representative pathogen was chosen for each pathogen class (bacteria (pathogenic *E. coli*), protozoa (Cryptosporidium), virus (rotavirus) and helminth (Ascaris)) with a load of 10^5–11^/person/day (see Supplementary Materials Table S1), with an infection prevalence ranging from 2% to 24% [61]. While the conceptual approach did not include population density, this dimension is considered important for predicting pathogen dilution factors, concentrations and exposure, and could be added into future versions of the model.

*Step 2. Calculate the flows*: In the absence of local data, to translate SFD per-population figures to volumes and flows, we used a standard assumption of 100 L/p/d water use with 30% discharged to the toilet [62]. Pathogen reduction by removal (i.e., containment, or filtration by soil) or inactivation (e.g., die-off, treatment) was estimated on the basis of available literature and expert opinion (see Supplementary Materials Table S2). Similar to the SFD process, working from left to right for each type of sanitation infrastructure, we used the SFD flow divisions to calculate the wastewater flow and pathogen load for each stream and we applied the system log_10_ reductions as pathogens passed through various treatment systems (septic tank, soil, etc.) or to account for die-off (i.e., in drains, groundwater, fields, etc.). A base flow and a dilution factor were used to account for additional inflows or dispersion when faecal waste enters the environment (such as discharging to local or community-scale drains, entering groundwater, or discharging to land or produce). While the preliminary dilution inputs were based on expected greywater flows, the dilution in the environment was recognised as a factor that requires understanding of local conditions and waste disposal practices. Our chosen approach for this illustrative case is described below as a part of Step 3. 

*Step 3. Calculate the pathogen concentration*: The pathogen concentration at each exposure point was calculated as the sum of pathogen loads divided by the flows discharged to that exposure point via the different pathways, calculated separately for each pathogen. These values were compared with pathogen concentrations noted in the literature (Supplementary Materials Table S6) and adjustments made to the dilution to ensure the calculated concentrations were of a similar order of magnitude to those noted in literature. Available literature for different pathogen classes and pathways was limited, for some pathways only *E. coli* data was available. Use of local data and better consideration of the hydrological factors would improve the modelling of dilution, while local environmental monitoring would be valuable to test the validity of inputs and approach.

*Step 4. Calculate the probability of illness*: Along with the calculated concentration for each pathogen at each exposure point, we used data from the literature to estimate exposure volume, frequency and exposed population (see Supplementary Materials Tables S3–S5). For each pathogen and each exposure pathway we calculated the dose, daily probability of infection and illness, annual probability of illness, and annual DALY per person; the annual DALY per person was multiplied by the percentage of population exposed to each exposure point for adults and children separately. The results across the four pathogen groups were then summed to estimate a “DALY per exposure point”, and summed to estimate the overall DALY for the base case. 

**Figure 4:**
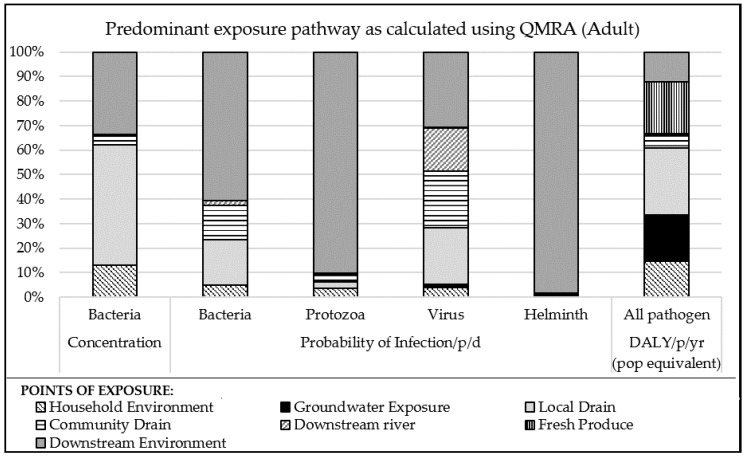
Illustrative outputs from the base case highlighting the importance of considering pathogens classes separately and calculating health risk in DALY to reveal priority points of exposure pathways.

Figure 4 presents one possible type of output from the model, which identifies the change in priority pathways depending on which of three parameters is considered: bacteria concentration, pathogen specific infection probability, and the resultant overall DALYs using QMRA. 

The results in Figure 4 highlight that inferences based on indicator bacteria concentration will result in different conclusions than inferences based on probability of infection or DALY. For example, in this case considering bacteria concentration alone could lead to the local drain being assessed as most important, and other important pathways are likely to be missed. The results of the probability of infection (considering dose and dose response) for the four pathogen types also demonstrates that the most important pathways varied for different pathogens, and highlights the importance of this aspect to the conceptual approach described in this paper. The health risk (based on illness and DALY ratios, exposure frequency and proportion of population exposed) demonstrates that there are contributions to this relative health risk through many different pathways, which help point to alternative sanitation options that could address these risks. The high proportion of DALYs from Groundwater and Fresh Produce exposure is influenced by the high assumed frequency of exposure (every day for Groundwater, 5 instances per week for Produce) and high proportion of the population exposed (35% population use groundwater, 65% consume fresh produce directly or indirectly irrigated or fertilised with faecal waste). The sensitivity of the model to exposure inputs should be assessed and highlights the need for local data, as is argued by Robb [7]. 

Due to the high infectivity of rotavirus and high excreted load, the probability of infection was high across all pathways (the reason these appear equally important in Figure 4). The subsequent step of calculating the annual probability of illness increased the probability to close to one (100% probability) and resulted in little change in the DALYs from viruses between improvement options. On review of the model results, we propose that the use of an annualised probability (maximum one illness per year) should be compared with a daily probability (each day has equal risk of illness) to understand the influence of the very high probability of annual illness on the results. 

**Figure 5:**
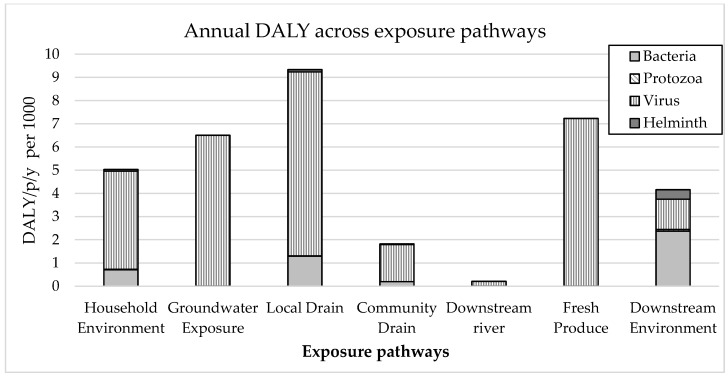
Illustrative output from the base case highlighting the significant pathways and relative contribution to health risk of different pathogens in each pathway.

Figure 5 shows an example model output of the contribution of each pathogen type to relative health risks for each exposure point. This output is illustrative only, and demonstrates that using such a model, it is possible to estimate the relative importance (in terms of health risk) of different pathogens at different exposure points, which can subsequently inform identification of appropriate sanitation options that best prevent or treat this situation.

Figure 5 demonstrates that virus is the predominant pathogen class, as expected due to the high rate of infection and high DALY per illness of the modelled rotavirus. There is some variation of the relative significance of other pathogens between pathways with bacteria contributing to DALYs at household environment and local drain exposures, while helminths are a risk in downstream environment. The high risk of Local Drain could be expected, given the base case involves 34% of the population’s faecal waste discharging directly to drains, including 21% direct from toilet without any pre-treatment, while also contributing to high household environment risk due to the reported frequent flooding of sewers and drains. The source of faecal waste that reaches Fresh Produce is predominantly from septic tank effluent only, and not sludge, due to very low sludge emptying rates (Table 2) which could explain the low levels of Helminth attributed diseases for Fresh Produce as Helminths are often reported to be more highly associated with the sludge than effluent.

The results shown in Figure 5 also align with other QMRA assessments which found the exposure to open drain caused the highest contribution to the annual health risk [5,34]. Other studies concluded open drains were the second highest contributor, after fresh produce [35] or after urban farmers working in wetlands [33]. The findings on the predominance of viruses in contributing to health risks also aligns with findings in the literature. In three recent field studies viruses and also bacteria were found to contribute to a greater portion of the overall health risk than protozoa and helminths [5,33,34]. 

These results are based on assumptions for the hypothetical base case and should not be interpreted as real findings for Dhaka or applied to other situations without further sensitivity testing and validation. The sensitivity of the model to the system log_10_ reduction assumptions should be further tested by considering the range of possible removal rates due to environmental and other variables. While the findings align with other studies, there is high uncertainty regarding the system log_10_ reduction assumptions due to a lack of available data for all pathogen classes. This required estimations to be made for some system log_10_ reductions, particularly for groundwater and for local drains. This reiterates the need to consider local conditions, as well as agricultural and consumption practices that affect log_10_ reduction, as well as further empirical research on the pathogen log_10_ reduction for all pathogen classes. These findings are illustrative only, presented to demonstrate how a model of this type can be used. 

*Step 5. Develop and test improvement options*: Alternative sanitation improvement options were developed on the basis of the most significant exposure pathways determined from the base case assessment. For the hypothetical case above, Figure 5 shows that exposure to local drains, household environment, groundwater and produce contribute the highest proportion of DALY for the adult population, therefore options that address these risks were developed and tested in the model. Specifically, six alternative sanitation options (including variations on these) were tested by revising the model set-up or inputs such as the flow division (i.e., reducing % sewer flows flooding), adjusting pathogen log_10_ reductions (i.e., improving treatment efficacy) or exposure to reflect actual system improvements. Changes in the DALY were then compared with the base case (Table 2). In Table 3 we focus on the relative changes in health risk. We do not show quantitative results due to the uncertainties associated with certain aspects of the model and input data limitations (see Section 4). 

The illustrative findings demonstrate the use of the model in understanding how changes in technical, service and exposure could affect different exposure points or overall relative health risk. The results highlight that: (i) improvements can shift pathogen flows and health risks downstream (1, 3b, 4a); (ii) downstream interventions may have minimal overall impact (5) if, as was the case in this hypothetical study, the health risks are predominately in the upstream local area; (iii) increasing emptying may have little health impact (4a) if the base case assumes no exposure to unemptied/stored septic sludge. The options that demonstrated a highest potential overall impact were: reducing exposure by covering drains (2), addressing wastewater conveyance issue to reduce flows in open drains and increase those flows reaching treatment systems (3d) and a non-traditional option (cover drains, reduce groundwater use and discontinue reuse of untreated sludge and wastewater for food production) to address the key exposure pathways (6). This example shows the value of the model in systematically identifying preferable interventions from a health risk perspective and assessing their relative improvement at specific exposure points and overall.

## 4. Discussion

This research has developed a conceptual approach to link microbiological theory with applied sanitation options assessment to inform decision-making based on public health risks. By extending existing sanitation tools and risk assessments, it provides a systematic approach to assess how sanitation services (or lack thereof) are contributing to health risks of varying magnitudes, and what the most appropriate solutions might be. Through a holistic approach that considers all types of sanitation improvements across the entire service chain, and multiple pathogens and exposure pathways, this concept provides a way forward in the face of data constraints that are typical in developing country urban contexts. The approach is complementary to efforts in the form of detailed empirical studies, which are very much needed, and has potential to improve the targeting of such studies. Overall, the approach provides a means by which available data can be integrated into a structured decision-support framework, providing a quantitative basis for relative comparison of sanitation options, and supporting a more robust consideration of public health benefits than existing qualitative approaches. This said, it should be clarified that the approach does not aim nor claim to provide robust quantitative predictions of absolute health risks, and indeed this is considered beyond scope; rather it provides a basis for relative comparison between alternative options. The illustrative application of the approach, using available data from one particular city context, demonstrates the benefits of this holistic approach in identifying the most significant exposure risks within an urban environment. It highlights the need to widen our consideration of health risks to exposures in the household, local community and downstream locations, and to consider how to prevent pathogen entry to the environment rather than only mitigate the consequences of their presence. 

This encourages a shift in thinking away from traditional end-of-pipe solutions such as centralised treatment plants at the city’s periphery (which focus on protecting the downstream environment), to target predominant exposure pathways and consider non-traditional solutions such as covering drains, conveying and treating effluent from on-site sanitation systems, and reducing sludge dumping in public areas or untreated faecal waste discharging to recreational urban waterways. Modelling the interconnected pathways ensures that when improving one aspect of the sanitation chain, the upstream and downstream consequences of this change are identified. This can help to assess whether proposed solutions are simply shifting the problem rather than solving it. Although tested only with a hypothetical example to date, such that the specific numbers should be taken with caution, the analysis indicates that, counter-intuitively, increasing sludge emptying or connecting toilets and septic tanks to sewer may not reduce the overall health risk in a city if the subsequent impacts on downstream systems and exposures are not concurrently considered. Conversely, it also highlights the limited health benefits of focusing only on a traditional centralised treatment plant if upstream exposure in the household and local community are not reduced.

It is clear that typical approaches to undertaking health risk assessment of individual sanitation technologies in isolation, without situating these within the city context or the broader service chain ignore the influence of resulting pathogen flows and exposures. These flows and exposures are critical to assessing priority health risks and locations. In addition, as we move towards a future of circular economy thinking and action in urban water and wastewater management, and increase recognition of the interlinked impacts of urban sanitation on safe water supply, the proposed approach assists in moving towards integrated thinking across water safety planning (WSP) and SSP processes and findings. To contribute to this, modelling can provide a next step to translate scientific knowledge into policy and practice for a holistic city-wide analysis across these domains.

A key challenge in developing the proposed approach was the trade-off between creating a framework simple enough to be applied in low resource environments and ensuring adequate treatment of the complexities inherent in linking pathogen flows with health risks. At this stage we propose that the conceptual approach and its application are primarily useful to guide researchers and sanitation experts (rather than practitioners or city governments) in furthering the global evidence base in this area. It assists the identification of key data gaps that prevent simplified modelling of urban sanitation contexts and the pathogen flows within them, and key areas that require further methodological development. In what follows, we discuss possible limitations of the proposed approach and offer suggestions for future research and practice.

### 4.1. Pathogen Data Gaps

The proposed approach distinguishes four pathogen classes and considers each separately due to their differing behaviour in the environment and their associated health risks. However, separate consideration of pathogen types also adds uncertainty, due to limited data for all pathogen classes on the typical log_10_ reductions within the different components of the sanitation chain and in drains, waterways, soil, etc. The GWWP is currently publishing up-to-date international reviews of pathogen specific, and treatment specific datasets. At the time of writing, relevant chapters on rotavirus were available; persistence of pathogens in sewage and other water types; waste stabilization ponds; and constructed wetlands. These reviews and expert consultation suggest a key data gap is the performance of sanitation systems as used in developing countries, particularly on-site sanitation systems and natural based treatment. 

A particularly significant gap is the lack of data on the apportioning of different pathogens between sludge and supernatant in on-site sanitation. Consulted experts suggested that it is likely that a greater proportion of helminths and protozoa will be present in sludge and a greater proportion of bacteria and pathogens in liquid, based on first principles considering the size and nature of these organisms; however, to date the division of pathogens in septic tank sludge and supernatant has not been ascertained through empirical research. 

Another uncertainty is the limited data on pathogen behaviour in drains and waterways, as available data focuses on inactivation, whereas removal by settling of helminths and protozoa in drains might also be expected. Pathogen data is often inconsistently reported, including treatment performance reported in % rather than log_10_ reduction [3], or is reported as a single value only, that ignores the likely differences in log_10_ reduction of different pathogen classes. Lastly, the validation of calculated pathogen concentrations in waterways was difficult since available data on pathogens in the environment is often limited to *E. coli*. Indeed, the GWPP concluded that there are significant data gaps in developing country contexts, in particular for protozoa or helminths in environmental waters [63]. 

Given these uncertainties, adoption of the proposed conceptual approach and future model developments should include sensitivity testing of the prevalence of disease, range of pathogen log_10_ reduction and division of pathogens in on-site systems. In addition, there may be other useful steps which can be taken in the absence of better pathogen data. Montangero [30], proposed the use of formal expert elicitation (e.g., the Delphi method) as an effective approach to understand the mechanisms and probability distributions for nutrient transfer coefficients in septic tanks, in the context of developing countries where data was scarce. Our view is that consolidating expert opinion in this domain, particularly concerning predicted system log_10_ reductions, is an important next step that should follow this research. In addition, it is possible that new technologies in DNA sequencing will improve the feasibility of quantification of pathogens in the environment.

### 4.2. Is QMRA Apropriate?

The model uses a QMRA approach to compare quantitative relative health risks of alternative options. QMRA was considered appropriate in this context due to the previous-mentioned benefits of considering the exposure, illness, infectivity and impact of different pathogens in one comparable health outcome, and also due to its status as a globally adopted tool endorsed by WHO. However, in expert consultation during this research, the following concerns were raised about the application of QMRA in this context. First, the underlying dose-response models, which typically rely on challenge studies undertaken with adults in developed countries, may not be transferable to developing countries and do not consider degree of immunity [64]. Second, exposure has been found to vary both between and within cities [7], and while there are increasing assessments in urban areas, the extent to which the data from one study can be translated to another location due to behavioural, geographical, seasonal variations is unclear [65]. Third, QMRA has traditionally been applied in low-pathogen environments (water supply and food) with single dominant pathways; this may call into question its validity in low-income poor sanitation areas where there are high concentrations of various pathogens and numerous exposure pathways [66]. Lastly, while this model used point estimates for inputs, a stochastic approach, such as Monte Carlo simulation, could be used in more complex QMRA models to better take into consideration the variability and uncertainty of inputs by including a probabilistic distribution of the range of values [56].

Given these concerns, in further developing the proposed approach, the sensitivity of the model to the prevalence of disease, dose response, exposure and DALY estimates should be tested, as this will confirm (or not) the relevance and appropriateness of a QMRA approach. It will also be important to avoid the potential misuse or misinterpretation of the results to predict or “claim” DALYs saved, rather than simply to compare options on a relative basis as intended. Beyond this, there may be alternative pathogen risk assessments methods that could be considered, such as health impact assessments [67], risk ratio of infection [22] or semi-quantitative approaches.

### 4.3. How Can Modelling Deal with the Complexity of Urban Sanitation?

Simplistic consideration of time, spatial and dilution aspects in the model may not be appropriate for some situations, particularly large cities with multiple or independent catchments or sanitation services. In the model presented in this paper, for simplicity, we chose to use an average concentration spread across the year, which ignores that some sanitation-related occurrences are intermittent, such as dumping sludge in drains or flooding. Additionally, pathogen loads may vary over time, with authors noting that *E. coli* is discharged daily whereas viral pathogens are likely to be intermittent [34]. As these would create short term concentration peaks and related hazards, they are likely to affect exposure and risk estimates. A time series dimension could be added (though would add complexity), and would also allow the consideration of high and low rainfall conditions. The approach to dilution (based on comparison with downstream concentrations data from literature) is a weakness of the model; better consideration of catchment hydrology is recommended. Spatial resolution could be valuable for considering different catchments or sanitation practices or services. This could be achieved by applying the model for sub-areas of similar conditions, such as flood prone areas or urban slums in comparison with high-income neighbourhoods and mapping the different results for different improvement options. While these aspects could be addressed through further model development, the intended purpose, required level of detail and data availability should be considered before increasing complexity. 

## 5. Conclusions

We have presented a proposed conceptual framework that enables application of existing scientific data and knowledge regarding pathogens and health risks to inform urban sanitation options assessment in developing countries. While existing tools and approaches have significantly advanced our understanding of sanitation service performance and pathogen exposure risks, this research identified gaps in their ability to predict faecal pathogen flows, relate exposure risks to existing sanitation services, and to compare expected impacts of improvements. Through a source-pathway-receptor approach, faecal waste discharge across the sanitation service chain was linked with potential pathogen exposure pathways in order to quantitatively compare improvement options. An illustrative application of the conceptual approach used a spreadsheet-based model to estimate exposure from different pathways for a hypothetical urban sanitation situation. Using QMRA to compare the relative effect on (DALYs of six sanitation improvement options, our results demonstrated that common traditional solutions for improving sanitation services (for instance building end-of-pipe treatment plant or developing faecal sludge emptying services) may be insufficient to address priority transmission routes that occur within urban areas. Rather, the results point to the need to consider a breadth of possible improvements along the sanitation chain, including non-traditional options of covering drains, preventing leakage and improving conveyance of both effluent and sludge to treatment plants. In essence, the modelling of pathogen flows demonstrated how some current sanitation “solutions” may simply move the health risk from one location to another, rather than solve it. The proposed approach is not intended to replace existing tools, but rather, it provides a framework that could integrate several existing tools and make use of existing scientific data. In doing so, the approach supports a structured consideration of public health in city-sanitation planning and has potential to help identify key data gaps and questions that require exploration through more detailed empirical investigation. Further development of the proposed conceptual approach on the basis of empirical research in selected case-study locations of varying conditions is suggested as a next step to address data gaps and continue to evolve the approach. 

## Figures and Tables

**Table 1:** Contributions and limitations of existing tools and approaches to estimate pathogen flows and health risks and inform decision making.

Approach	Description	Assessment of Approach or Tool (in Relation to Estimating Pathogen Flows and Health Risks to Inform Decision-Making)	
		Contributions	Limitations
Shit Flow Diagram (SFD) [25,26]	- Advocacy and decision-support tool that assesses the fate of excreta in urban areas based on secondary data and stakeholder interviews or primary field research - Produces a graphic representation of the proportion of population’s excreta that is considered “safely” or “unsafely” managed in terms of service outcomes along the sanitation service chain - Similar: Urban Sanitation Status Index [27], and Faecal waste flow calculator [28]	- Relatively simple diagram for identifying the major service failures and advocating improvements across the service chain - Considers multiple flow paths of both solid (sludge) and liquid (effluent) waste - Reports available from 50 countries - Standardised definitions of sanitation types - Aligned with Sustainable Development Goals	- The approach does not specifically identify health/pathogen hazards as the approach does not consider the volume of excreta flows, or pathogen concentrations or exposure, and hence it is not a risk assessment tool - Definition of “safe” is based on achieving a service standard as agreed with city stakeholders rather than “safe” in terms of actual or relative health risk
Material Flow Analysis (MFA)	- Systematic assessment of stocks and flows of material within a defined system in space and time [29] - Applications for sanitation include assessment of nutrient flows [30] and *E. coli* [31]	- Quantifies flow and load considering changes (i.e., treatment) and interconnected pathways, can therefore quantify the effect on the entire system if one part is changed - Potential for varied scale and complexity of analysis	- Quantifies the system inputs and outputs but not the impact/consequence or spatial aspects - Limited data in developing countries, however there is potential to use “expert judgement” to fill data gaps [30]
Quantitative Microbial Risk Assessment (QMRA)	- Method to quantitatively assess scientific data in the context of estimated health outcomes due to the potential or actual exposure to infectious microorganisms [32] - Four step risk assessment process: hazard identification, dose–response assessment, exposure assessment and risk characterization	- Considers the difference in pathogens’ infectivity and health effects and the different frequency, volume and proportion of population exposed through different pathways - Increasingly applied in low-income countries, including field-based studies on exposure from wastewater reuse or drainage channels [5,21,33,34,35]	- Often based on limited samples of indicator organisms due to cost and difficulty of measuring pathogens in low-income countries - Does not generally include systematic analysis of how or where pathogens enter the environment, and therefore proposed improvements are often limited to reducing exposure through behaviour change or physical barriers rather than preventing pathogen entry to the environment - Other potential limitations of QMRA included in the Discussion section
Sanipath [7]	- Assesses exposure to faecal contamination in urban neighbourhoods based on questionnaires, field surveys and environmental microbiology samples - Produces an exposure risk profile (percentage population exposed and monthly average *E. coli* dose) for multiple exposure pathways associated with inadequate sanitation	- Detailed assessment of behaviours to understand site-specific child and adult exposure including dose and frequency - Data available on environmental contamination and exposure behaviours in five cities - Findings in Ghana highlighted widespread pathogen contamination in public areas, with high *E. coli* concentration in drains and highest exposure risk in fresh produce	- Exposure is based on *E. coli* only, which may not be representative of other pathogen types - Difficult to compare findings (i.e., Prioritise between high dose and low % population exposed vs low dose and high % population) - Results are not linked to the source of pathogens; proposed improvements therefore focus on exposure risk mitigation rather than prevention of pathogens entering the environment - In environments with high pathogen concentrations from multiple sources, limited sampling may not capture all risk pathways
Sanitation Safety Planning (SSP) [24]	- Risk assessment approach to systematically identify and manage health risk along the sanitation chain and guide investment based on health risks - Participatory approach to include actors from different sectors to identify risks and agree on improvements and regular monitoring - Applicable to all sanitary systems, however it was developed for the implementation of the Guidelines for Safe Use of Wastewater, Excreta and Greywater	- Draws on local knowledge to identify health hazards and exposure pathways - Promotes a multi-barrier approach with a focus on achieving pathogen log reduction - Step-by-step guidance to identifying and assessing hazards and highlighting the numerous pathways of exposure to various user groups	- Risk assessment of likelihood and severity is subjective and may not be informed by sufficient evidence - Control measures focus on disease transmission routes rather than source of pathogens in the environment due to a strong focus on wastewater and excreta reuse in agriculture - Difficult to quantify effectiveness of control measures [36]
Rapid Participatory Sanitation SystemRisk Assessment (RPSSRA) [20]	- Draws on local community knowledge and their perception of their environment to derive risk scores based on a set of pre-defined indicators - Participants rank the risk for their neighbourhood from a set of defined conditions for 14 indicators	- Identifies local behaviours, status of services and contextual factors that influence exposure risks - Provides a rapid and resource-efficient assessment of behaviours and exposure with results from small-group discussions validated with detailed survey	- Risks are not health focused and the predefined indicators are site specific and subjective - Traffic light assessment may limit differentiation between exposures (i.e., most indicators were ranked high risk)
BORDA risk mapping [37]	- Guide to planning sanitation based on mapping existing services, environment and health data to identify priority and challenging areas - Water, wastewater and waste options identified based on population density, road width and income	- Spatial analysis of priority locations due to multiple hazards; informs option selection based on physical and economic factors - Applied in Dar es Salaam, Tanzania	- Requires detailed spatial data - Assumes risks to be higher where there are overlapping hazards but does not consider exposure, which limits health risk assessment
Technology options assessments (various)	- The EAWAG Sanitation Compendium [1] presents a complete overview of individual technologies, their advantages and disadvantages, and how they can be linked together in a systems approach; although no explicit approach for comparison or relating to existing pathogen flow situation in an urban environment is provided. Various other tools compare individual technology options through an indicator approach, including NESTAFF [38] and TAF [39]	- Detailed description of individual technologies, including their performance against criteria or indicators	- Health aspects, if included, are typically qualitative and limited to a broad assessment of whether an exposure or health risk exists - Do not typically consider how proposed solutions relate to or build from existing sanitation conditions and services, or recognise that a single sanitation solution in such contexts is unlikely
Microbial Exposure and Health Assessments in Sanitation Technologies and Systems [22]	- Assessment of the health risks of each technology in the EAWAG Sanitation Compendium based on risk ratio for diarrhoea infection or helminthiasis	- Assessment of system performance to remove all four classes of pathogens - Considers system efficiency, robustness, and different exposure pathways and risks for worker, farmer, community	- Unclear how the risk ratio (infection per 10,000 p/y) is calculated - Focus on individual technologies rather than the combined risk of a system and how it is influenced by local context
Saniplan [40]	- Excel-based decision support tools, developed for India, considering infrastructure and service improvements and financial planning	- Considers entire sanitation service chain and based on current service performance (access, service quality, efficiency, finance)	- Suggested improvements based on comparison of key performance indicators (e.g., % households with improved sanitation) rather than health risks
Sanitech [41]	- Tool for assessing options in Indian cities, based on spatial data, physical constraints and cost	- Comparison based on cost, coverage and environmental treatment performance	- Health risks not considered in selection or comparison of improvement options
Citywide planning tools (various)	- Various planning tools focus on the steps for implementing planning, including Community-Led Urban Environmental Sanitation Planning (CLUES) [42], Sanitation 21 [43], Performance Assessment System (PAS) [44], Citywide Sanitation Strategy [45], City Sanitation Plan [46]	- Highlight the importance of a participatory approach and of considering local conditions and service status, typically along entire sanitation service chain - Considers other services (drainage, waste) - Governance and finance focused	- Health often not included in criteria used for comparison of options (Citywide Sanitation Strategy, PAS) - Guidance typically does not inform decision on different technical solutions or the extent to which different options achieve overall objectives

**Table 2:** Inputs to model for Dhaka (Bangladesh) base case.

**Containment**		**Sewer**	**Drain**	**Septic Tank (ST)**	
	Toilet discharge	25%	21%	54% *	
	ST supernatant portion of ST flows			50%	
	Discharge of septic tank supernatant:	3%	49%	2% (no outlet)	
		Conveyance as per toilet to sewer or drain		To ground/groundwater	
**Conveyance**		**Sewer**	**Drain**	**Septic Tank Sludge**	
	Sewer/Drain overflows	25%	25%	Sludge emptied	12%
	Sewer/drain leakage **	2%	2%	Not-emptied/stored on-site	83%
	Continues in sewer/drain	73%	73%	Overflow to ground	5%
**Disposal**	Treatment	43%	1%	Local drain	73%
	Waterway	52%	89%	River	23%
	Agriculture reuse **	5%	10%	Land-not used	2%
				Land-reuse	1%
				Treatment	1%
**Treated reuse**	Waterway	90%		River	20%
	Agriculture reuse **	10%		Land-not used	75%
				Land-reuse	5%

Note: This table is also shown as a tree-diagram in Supplementary Materials Figure S1. * The Dhaka SFD report [60] states that it does not include pit latrines due to data only reporting septic tanks, although it recognises that it is unlikely these are all standard septic tanks, particularly in low income areas. ** Leakage and reuse have been added for the purpose of illustrative application. The values for the percentage of systems emptied is based on previous World Bank estimate for the SFD [59], rather than the assumption in the Water, Engineering and Development Centre (WEDC) 2015 report that systems with an outlet do not require emptying.

**Table 3:** Change in DALY per person per day from base case (based on exposure frequency and proportion of population exposed for Adults).

Improvement Option (Refer to Base Case in Table 2, and Detailed of Options Described in S7)	Household Environment	Groundwater	Local Drain	Community Drain	Downstream Waterway	Fresh Produce	Downstream Environment	Total	Explanation of the Results.
1a. Reduce leakage from sewer and drain into groundwater (as 25% population assumed to use groundwater daily for drinking)	0%	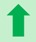	0%	0%	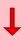	0%	0%	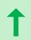	A very small change in leakage flows from sewer and drain (2% change) resulted in an overall reduction in health risk, despite a slight increase in risk in relation to downstream waterways
1b. Reduce groundwater use for drinking by half by providing an alternative water supply	0%	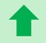	0%	0%	0%	0%	0%	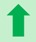	The health risk associated with the groundwater pathway was significantly reduced. Groundwater risk reduction by providing an alternative water supply may have a greater positive impact than reducing groundwater pollution (1a).
2. Cover local drains	0%	0%	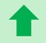	0%	0%	0%	0%	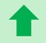	Covering drains reduced exposure and related health risks through this pathway, and resulted in a major overall reduction in health risk due to significance of this pathway.
3a. Toilet and septic tank effluent to sewer (not drain)	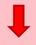	0%	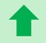	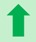	0%	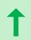	0%	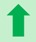	Reduction of faecal flows to open drain reduces subsequent exposure at local and community drains, but moves pathogen flows so increases risk at household due to no improvement in the sewer overflow/flooding.
3b. Improve conveyance (reduce flooding and leakage)	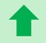	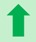	0%	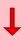	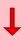	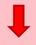	0%	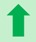	Reducing flooding and leakage reduces health risk in the immediate household area and in groundwater, although without improving treatment there was a slight increased downstream risk in waterways and food produce.
3c. Increase sewer discharge that reaches treatment plant	0%	0%	0%	0%	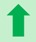	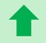	0%	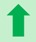	There is a reduced health risk associated with downstream waterways and food produce, however the overall health risk reduction is medium, as this option fails to address risks associated with upstream pathways.
3d. Improve wastewater conveyance (3a, 3b and 3c)	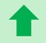	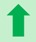	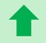	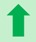	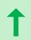	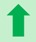	0%	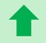	Addressing all issues with improved conveyance reduced the health risk associated with all pathways and results in a major overall reduction in health risk.
4a. Increase sludge emptying	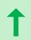	0%	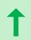	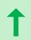	0%	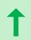	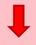	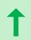	Increasing sludge emptying frequency has the potential to increase risk, as unemptied (stored) sludge was assumed to have no exposure. While emptying benefits the septic tank effluent quality, (i.e., reduced pathogen hazard in this effluent), without also improving conveyance and sludge treatment the results show a significantly increased health risks in the downstream environment, so overall there was only a small reduction in health risk.
4b. Increase sludge emptying and its delivery to sludge treatment plant	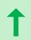	0%	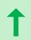	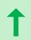	0%	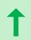	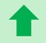	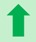	Increasing emptying and delivery to treatment reduced health risk in the downstream environment, however the population exposed was small so the overall reduction in health risk is small.
5. Improve faecal sludge treatment and wastewater treatment	0%	0%	0%	0%	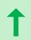	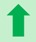	0%	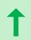	Traditional treatment solution that only addresses downstream exposure pathways. This option only resulted in a small reduction in overall health risk since emptying and conveyance were unchanged.
6. Cover drains, reduce groundwater use, discontinue reuse of untreated sludge and wastewater for food production	0%	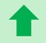	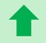	0%	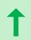	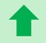	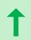	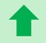	A non-traditional solution that addresses the key exposure pathways and resulted in the highest overall reduction in health risk compared to the base case.
Legend:	Change in DALY pppy from base case	Improvement in health risk	Worsen health risk	Relative change	No change	Small (±1–3%)	Medium (±4–13%)	High (>14%)	
					0%	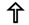	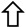	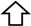	

## Supplementary Materials

The following are available online at http://www.mdpi.com/1660-4601/15/2/181/s1, Table S1: Pathogen inputs, Table S2: Pathogen log_10_ reduction assumptions, Table S3: Steps in the QMRA Calculations, Table S4; Exposure volume, Table S5. Exposure frequency and exposed population, Table S6: Environmental data use to validate the range of concentration at exposure points and to adjust the dilution, Table S7: Option modifications as analysed in the model and referred to in Table 2 of the main text, Figure S1: Inputs to model for Dhaka Bangladesh Base Case.
