# Supplementary material for: Faecal Pathogen Flows and Their Public Health Risks in Urban Environments: A Proposed Approach to Inform Sanitation Planning
Source: Int J Environ Res Public Health. 2018 Jan 23;15(2):181. doi: 10.3390/ijerph15020181 (PMC5858256; doi:10.3390/ijerph15020181)
Supplement: Supplementary file 1 [file ijerph-15-00181-s001.pdf]

Supplementary Material: Faecal Pathogen Flows and Their Public Health Risks in Urban Environments: A Proposed Approach to Inform Sanitation Planning

Freya Mills, Juliet Willetts, Susan Petterson, Cynthia Mitchell and Guy Norman

1. Model Inputs and Assumptions

The pathogen inputs used in the illustrative model are summarised in Table S1.

Table S1. Pathogen inputs.

Inputs Per Pathogen Group	Bacteria	Protozoa	Virus	Helminth
Reference pathogen	Pathogenic *E .coli*	Cryptosporidium spp.	Rotavirus	Ascaris lumbricoides
Faecal concentration infected individuals.	10^8 org/g·wet faeces	10^6 org/g·wet·faeces	10^8 org/g·wet·faeces	10^5 org/g wet faeces
Faecal mass excretion per day		243 g·cap^{-1}·day^{-1}		
Prevalence	6%	8%	2.0%	24%

1.1. Selection of Reference Pathogens

According to the reference pathogen principle, pathogens from each microbial group (bacteria, viruses, protozoa and helminths) are selected that are of local regional significance and are assumed to be a conservative representative of its group. Consideration is also given to the available data to quantify the occurrence, persistence, infectivity and disease burden. For the present study representing an urban setting in the developing context, the following reference pathogens were selected:

- Bacteria: Pathogenic *E. coli* (specifically Enterohaemorrhagic *E. coli*)
- Protozoa: *Cryptosporidium*
- Virus: Rotavirus
- Helminth: *Ascaris Lumbicoides*

1.2. Concentration in Faeces of Infected Individuals

1.2.1. Bacteria

Feachem [1] reported that ETEC concentration in faeces to range from 10^8–10^9 per gram, however no citations were given. A value of 10^8 was selected.

1.2.2. Viruses

De Silva [2] reviewed the data on the concentration of rotaviruses in faeces and reported that persons can shed concentrations of 10^{10} to 10^{12} of virus per gram. However as virus excretion varies over the course of an infection [3,4], the reported concentrations are most likely peak excretion and therefore a lower value of 10^8 was applied to be representative of the overall loading.

1.2.3. Protozoa

Medema [5] reported that *Cryptosporidium* concentration in infected individuals to range from 10^5 to 10^7 oocysts per gram citing Chappell [6], a value of 10^6 oocysts per gram was selected.

1.2.4. Helminths

Feachem [1] reported that individuals excrete up to 300,000 eggs per gram of faeces, however no citations were given. 10^5 eggs per gram was selected as a starting point for the model.

1.3. Prevalence

Platts-Mills [7] investigated the pathogen-specific burdens of community diarrheoa in children (< 2 years) in developing countries covering eight contexts in Asia, Africa and South America. A total of 31,628 stools were tested of which 7318 were from symptomatic children. Prevalence of positive detection of pathogens was taken as an estimate of the different levels pathogen prevalence in the community. Prevalence of pathogenic E. coli was based on prevalence of enterotoxigenic *E. coli* (St-ETEC); viral prevalence based on Norovirus GII; and protozoa on *Cryptosporidium* (noting that *Giardia* was much more prevalent than *Cryptosporidium* at around 30% in 12–24 month old children). Percentages in Table S1 are approximate as the numbers were read off a low-resolution graphic (Figure S1), however for the purpose of this illustrative case this was considered suitable. In addition, the prevalence of pathogens is likely to be higher amongst children in comparison to adults, and therefore the values selected are an overestimate for a mixed age population. In reality, the prevalence of different pathogens will vary between communities and also within a given community over time. The purpose of the modelling tool would be to explore this variability, rather than simply include it as a fixed value. As a starting point for Helminths, Pham-Duc [8] reported an *Ascaris Lumbicoides* prevalence of 24%, amongst agricultural communities in northern Vietnam (n = 1425). No representative values more suitable to the urban context were identified.

1.4. Faecal Excretion Per Day

Rose [9] reviewed the amount of faecal material excreted per person per day. For low income countries (n = 17) the mean was 243 with a range of 75–520 $g \cdot cap^{-1} \cdot day^{-1}$, noting that this is the variability in the mean, individual variability would indeed exceed this range. Diarrhoea has an impact on stool production, structure, form and composition, leading to much higher faecal mass generation and water content. A point value equal to the mean of 243 $g \cdot cap^{-1} \cdot day^{-1}$ was selected.

1.5. Pathogen log_{10} Reduction

Inputs to the model for each pathogen class to account for the reduction in pathogens from excreta to exposure, including formal treatment systems and the pathogen reduction during conveyance or discharge to the environment. The range of data shown in Table S2 was sourced from a range of literature, with greater data availability for traditional treatment systems (i.e., wastewater or sludge treatment plants) than for conveyance processes (flows in drains, sewers, groundwater). Key limitations of the data were reference to log_{10} reduction without differentiating between pathogens (i.e., die off after irrigation 0.5–2 log_{10}), not distinguishing whether the removal referred to the liquid or sludge components (particularly for septic tank and sludge treatment) and often only providing data for some pathogen classes. Reduction in soil, fresh produce and groundwater varies significantly depending on the local conditions, disposal or irrigation practices, and selected log_{10} reduction should be based on local data where available.

This table was developed for the purpose of the preliminary model testing and many assumptions have been made. The literature analysis used for this table was not extensive in the knowledge that the Global Water Pathogen Project was synthesising existing research in detail, and although not finalised at the time of writing, is expected to greatly inform this table. The numbers in brackets have been adopted in the preliminary model presented in the paper, however it would be important for further development of the model to test the sensitivity to the range of reductions.

Table S2. Pathogen log$_{10}$ reduction assumptions.

System	Bacteria *E. coli*	Protozoa *Cryptosporidium*	Virus *Rotavirus*	Helminth *Ascaris*	Source
Septic tank effluent, regularly emptied	0–2 (1)	0–2 (2)	0–2 (0.5)	<1 (0.8)	Operating as designed. [10,11]
Septic tank effluent, not regularly emptied	No data (Ass. 0.8)	No data (Ass. 1.5)	No data (Ass. 0.4)	No data (Ass. 0.6)	No data, assumed lower than regularly emptied
Septic tank sludge, assumed stored for 3–5 years	No data (est. 1–4, 3)	No data (est. 0–2, 1)	No data (est. 1–2.5, 2)	No data (est. 0–1.5, 1)	No data. Estimated from data on pathogens in fresh faeces compared with emptied sludge concentration [12]
Primary wastewater treatment (settling)	0–1 (0.3)	0–1 (0.5)	0–1 (0.3)	0–1 (0.5)	Primary sedimentation [11]
Secondary wastewater treatment	3–6 (6)	1–4 (4)	2–4 (4)	1–4 (3)	Waste stabilization ponds: Maximum for optimal function with 3–4 ponds in series [13,14]
Primary faecal sludge treatment—sludge drying beds	1 to 6 (2.7)	NA (assume 2)	NA (assume 1)	1 to 3 (1.6)	Sludge settling pond, bacteria and helminth [10] Protozoa and virus assumed based on relative survival in soil.
Secondary sludge treatment: co-composting	2 to 6 (5.5)	1.8 to 6 (2.5)	2.5 (2)	1 to 2 (2)	[10]
Groundwater and soil filtration—fine loamy soil, <10 m·well	T90 *E.coli* 1–25 days (est. 4 log$_{10}$)	T90 34-200 (at 4 degs, faster when warmer) (est. 6 log$_{10}$)	T90 31–120 days 3 log$_{10}$10 days (est. 3 log$_{10}$)	No data (est. 5 log$_{10}$)	Time for 1 log$_{10}$ reduction in groundwater 15–20˚C Rough estimate also considered filtration of helminth and protozoa due to size and biological processes [15]
Open drain (light)	*E.coli* 2–5 days (est. 0.2)	No data, faster than dark sewer (est. 0.03)	1.7–35 days (all virus) (est. 0.04)	Years/many months (est. 0.006)	Time for 1 log$_{10}$ reduction at 20˚C [16]. Estimated log$_{10}$ reduction based assumed time for low flow in 10km. Data appears not to consider removal by sedimentation.
Sewer (closed, dark)	Less in dark, *E.coli* 4–11 days (est. 0.1)	38–86 days (est. 0.02)	22–115 d (Norovirus 19–49 d Poliovirus (est. 0.02)	No data (est. 0.006)	
Soil–Die off in the environment (assume 2 weeks storage)	20–70 days (est. 1)	10–20 days (est. 2)	20–100 days (est. 0.8)	Many months (est. 0.5)	Time for 1 log$_{10}$ inactivation at 20˚C [1]. Estimated log$_{10}$ reduction after 2 weeks storage.
Die off in produce before harvest (after 1 week)	(est. 3)	(est. 4)	(est. 2)	(est. 1.5)	0.5–2 log$_{10}$ reduction per day [11], variation across class estimated from time for 1 log$_{10}$ reduction for produce [1]

1.6. QMRA Calculations

Based on the standard QMRA methodology [17], the following the steps detailed in Table S3 below were applied to the calculated pathogen dose at each point of exposure for adults and children separately. Dose-response models were used to estimate the infection based on exposure to each pathogen, applying a beta Poisson model for *E.coli*, cryptosporidium and rotavirus [18,19,20] and an exponential model [17] with $r = 1$ for Ascaris. The illness infection ratio was assumed to be worst case of 1 for Pathogenic E. Coli and Ascaris [21] and the values for rotavirus and cryptosporidium were based on WHO Drinking water Guidelines [22]. While the ratio of DALY per infection can be estimated based on local probability estimates for the severity of diarrheal diseases and life expectancy, for this model of a hypothetical situation values were used from literature [23,24].

Table S3. Steps in the QMRA Calculations.

Step	Equation
Concentration	Calculate the load and flow along each flow pathway and sum the loads and flows coming to each exposure point to calculate the concentration of each pathogen.
Dose per person per day	d = Concentration × Volume consumed
Probability of infection (per person per day)	Beta Poisson model for Bacteria, Virus and Protozoa<br>Probability of infection (P_{inf}) = $1 - [1 + (d/\beta)]^{-\alpha}$<br>Dose response models and input values were:<br>Bacteria: $\alpha = 0.373$, $\beta = 39.71$ [20]<br>Protozoa: $\alpha = 0.115$, $\beta = 0.176$ [19]<br>Virus: $\alpha = 0.167$, $\beta = 0.191$ [18]<br>Exponential model for Helminth ($P_{inf} = 1 - e^{-r.dose}$) assume r = 1
Probability of illness (per person per day)	= probability infection × illness-infection ratio<br>Illness infection ratio for bacteria and helminth = 1, protozoa 0.7 and virus 0.5 [22]
Probability illness (per person per year)	= 1− (1-Probability daily illness) ^{N} where n = days per year exposed
DALY/person/year	= Annual probability of illness × (DALY/illness)<br>DALY/illness: E. Coli 0.0547, Cryptosporidium 0.00147 [23], Rotavirus 0.026 [21], and Helminth 0.0082 [24]
DALY/person/year (considering population exposed)	= DALY/person/year × percentage of the population exposed to each pathway

1.7. Exposure Assumptions

For the hypothetical case study, exposure inputs were based on literature from recent sanitation health risk assessments in similar low-income neighbourhoods or developing countries. This includes the estimated dose or volume consumed at each exposure point (detailed in Table S4), the expected frequency of exposure events per year (Table S5) and the likely proportion of population exposed to this pathway (Table S5). The data used in these references comes from both in-field surveys and literature. Due to the variability of all aspects of exposure on local conditions and behaviour and varying within cities and across seasons, Robb [25] argues for the use of local data, which have been determined in previous studies by questionnaires and field surveys and focus groups [25,26].

Table S4. Exposure volume.

Exposure Pathway	Behaviour	Volume Consumed L/p/d		Comments/Source
		Adult	Child	
Household Environment	Hands/fomite	0.005	0.005	Ground, hands fomite: soil from open space 5 mL (dose 9.14 × 10^3 *E.coli*) [27]
Groundwater Exposure	Drinking supply	0.500	0.500	Assume main supply for low income areas: 0.5 L·pppd for slum areas [27]
Local Drain—small/shallow near house	Playing (kids), flooding (adults)	0.005	0.003	Unintentional consumption during flooding: 10–30 mL [28] or 1 mL [29] Unintentional consumption during play: 1–5 mL [27–29]
Community drain/canal	Swimming/bathing, secondary water source.	0.010	0.020	Unintentional consumption due to recreation: Adult 0.016 L and child 0.037 L (does not include washing or bathing or occurring at different scales). [30]
Downstream river—receiving waterway	Swimming/bathing, secondary water source.	0.010	0.020	
Fresh Produce	Consumption	0.001	0.001	Serving of lettuce and other fresh produce: 1–5 mL (assume 1 serving per exposure) [30]
Downstream Environment	Accidental ingestion-adult farmers, children playing	0.005	0.005	Accidental ingestion: 1–5 mL farmers assuming some personal protection (gloves), or entrepreneur spreading sludge [28,31] Child playing at sludge storage 5mL [31]
Stored	No exposure	0.000	0.000	No exposure pathway

Table S5. Exposure frequency and exposed population.

Excreta Ingestion from Exposure at:	Events Per Year (N)		Proportion of Population Exposed		Comments and Source
	Adult	Child	Adult	Child	
Household Environment	12	12	35%	35%	Ground, hands fomite—assume once per month for slum population [27]
Groundwater Exposure	365	365	25%	25%	25% population use groundwater [32]
Local Drain—small/shallow near house	6	12	35%	35%	Adults exposed during flooding 1–6 times per year and children exposed daily to every 4 months due to playing in drains [27,29]
Community drain/canal	6	6	7%	18%	Assume 50% adult slum population bathing/washing in drain. Assume 50% child slum population swim. 6/year [28,29]
Downstream river/waterway	6	6	1%	1%	Swimming 6/year and 1% from a suvey in Kampala [33]
Fresh Produce	140	140	65%	65%	General produce 140 d/year [30] and 65% population exposed based on Sanipath findings [25]
Downstream Environment	52	1	5%	2%	Accidental ingestion by urban farmers (5% population) once a week. Children playing in field (2%) once a year [27–29]

1.8. Data for Validation

For a preliminary validation of the concentration of pathogens at the exposure point, the calculated pathogen concentrations at various points in the model were compared with literature to confirm the results were within the range of previously reported values. The data in Table S6 was developed for the purpose of developing the model and was drawn from a brief literature review (as this was not the focus of the research) which does not consider the full range of possible values when all variables are considered. As for Table S2, it is expected that this table would be significantly improved when the Global Water Pathogen Project is finished which will include detailed summary of the pathogen concentrations for the four considered pathogens (not yet available at the time of writing). As highlighted below, there is limited data on all pathogen classes for all pathways, with

most research reporting *E. coli* concentrations only. For this reason, the ratio to other pathogens was calculated to provide a potential range for comparing calculated values. However it is recognised that these ratios would be expected to vary depending on the pathway and treatment stage.

Table S6. Environmental data use to validate the range of concentration at exposure points and to adjust the dilution.

Flow/Path	Concentration Pathogens	Source/Comment
Septic tank effluent	*E. coli*: 1.2–2.2 × 10^{6}/100 mL	*E.coli*: Pang 2003, Dubber 2014 from [34]
Septic tank sludge	*E. coli*: 10^{1-8} CFU bacteria /kg·TS<br>Protozoa 10^{4-6}/kg·TS, Virus 10^{3-5}/L,<br>Helminth: 70–735/g·TS	Concentration of pathogens in primary sludge Table 48.9 Assume TS: 12,000–35,000 mg/L [12]. Helminth ova per gram TS, developing countries [35].
Local drain	*E. coli*:10^{8} CFU/100 mL<br>Rotavirus Open stormwater drain: mean 1.66–29.8/mL	*E. coli* from Accra (Ghana) for open drains [25,29]
Community drain	*E. coli*: $10^{5.4-6.9}$ CFU/100 mL, Rotavirus 0.34–8.85/mL	Sampling in urban slum in Uganda found mean value for stormwater drain and greywater drains [27,33] Virus from Uganda open drain [27]
Local Sewer /inflow to WWTP	*E. coli*: 10^{6-8} organisms/100 mL<br>Cryptosporidium 10^{0-4}/L, Rotavirurs 10^{2-5}/L,<br>Ascaris 10^{0-3}/L	*E. coli* [36], Other: [11: Table 3.2]
WWTP discharge	*E. coli*: 10^{3-6} CFU/100 mL	Volume 2 Chapter 6 includes stabilisation and trickling filter [12]
Treated sludge	Protozoa 10^{1-6}g/kg·TS, Virus 10^{1-3}g/kg·TS, Helminth 10^{1-4}/kg·TS	Dewatered and digested sludge [12] Bacteria data only given for extended aeration and was higher than inlet.
River or downstream waterway	*E. coli*: 10^{4}–$10^{6.6}$ CFU/100 mL, Rotavirus: $10^{-0.1 \text{ to } -0.01}$/100 mL, Ascaris $10^{0.48-0.6}$/L	*E. coli*—Uganda water channel, wetland, swimming lagoon [29,33] Virus and Helminth—Drain and stream water reused in agriculture [37]
Household environment	*E. coli*: $10^{5.4-6}$ CFU/100 mL	Samples from soil in open space; playground for children [28] and unintentional ingestion flood water [29]
Groundwater	Dug well: Fecal coliform levels between 10^{5}–10^{6} CFU/mL	Dug wells in a neighbourhood with septic systems (Gondwe 1997, referred to in [34].
Downstream environment	*E. coli* $10^{2.3}$/g·soil, $10^{5.6}$/100 mL irrigation water	Contamination of irrigation water and soil, Accra (Ghana) [38]
Fresh produce	*E. coli* 0.64 to 3.84 $\log_{10}$/g·produce [38]<br>Helminths—10^{3}/L and 10^{6}/L faecal coliform [39]<br>Rotavirus $10^{-0.7}$/100 g·wet·weight, Ascaris: $10^{3.8-5.7}$/100 g·wet·weight [37]	*E. coli*—consumption 52–102 g/salad week<br>Helminth and Faecal coliform: Mean contamination on produce at farmgate fed by stream water in South Africa [39]<br>Virus and Ascaris wastewater irrigated lettuce in Accra (Ghana), values at Farm [37].
Ratio of pathogen concentration to *E.coli*	*E. coli* O157: H7 7.6 × 10^{-4} to 10^{-2}. Cryptosporidium 5.5 × 10^{-7}, Rotavirus 5.5 × 10^{-6}, Ascaris 10^{-6} per *E.coli*	Used to compare *E. coli* concentration with other pathogens when data unavailable. [11,28,40]. However it is recognised that these ratios would not remain constant across the various pathways and treatment stages.

1.9. Sanitation scenario inputs - base case and improvement options

The illustrative case was primarily based on data from the Dhaka Bangladesh SFD report [32] with the division of wastewater flows between sanitation services shown in Figure S1, which is a visual representation of the data shown in Table 2 in the main report. Alternative sanitation improvement options were then developed on the basis of the most significant exposure pathways determined from the base case assessment. The options were tested by revising the model set-up or inputs such as the flow division (i.e., reducing % sewer flows flooding), adjusting pathogen $\log_{10}$ reductions (i.e., improving treatment efficacy) or exposure to reflect actual system improvements.

The details of these changes are shown in Table S7, with the results of the options analysis presented in the main report Table 3.

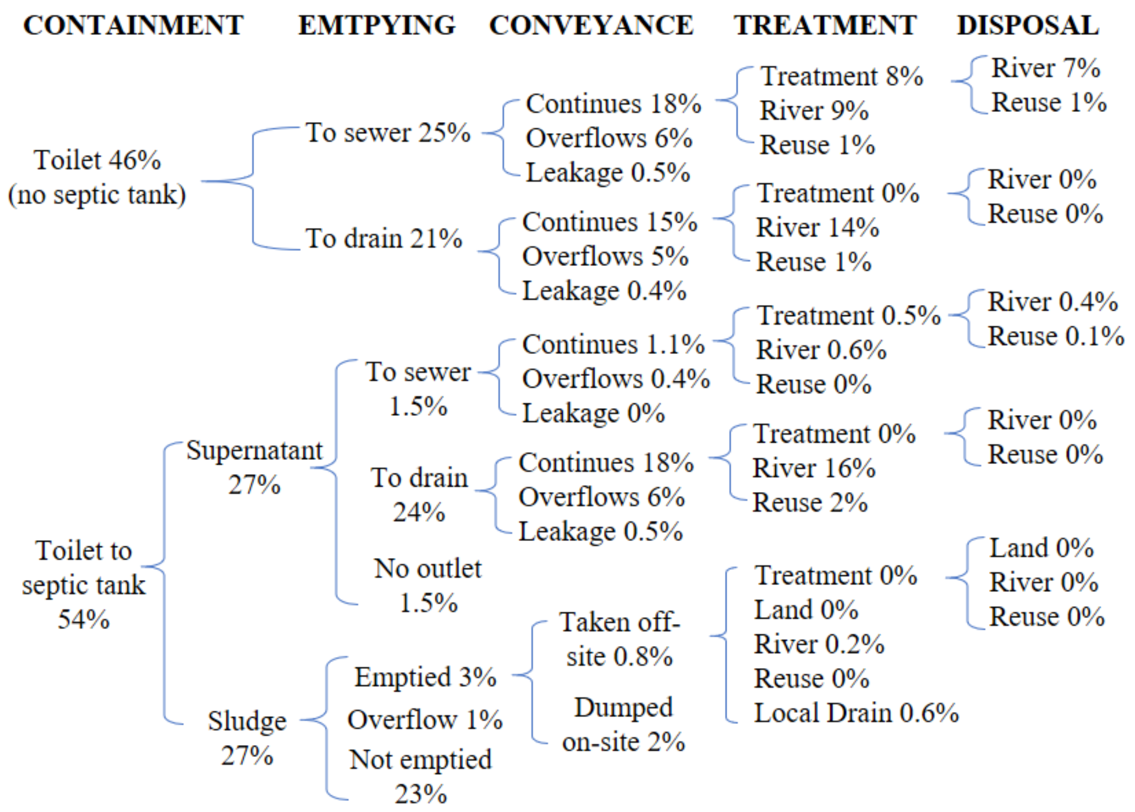

Figure S1. Inputs to model for Dhaka Bangladesh Base Case (alternative to Table 2 in main text).

Table S7. Option modifications as analysed in the model and referred to in Table 3 of the main text.

Improvement Option	Changes made to the Base Case Inputs to Model Improvement Options
1a. Reduce leakage from sewer and drain	Leakage from sewer and drain: based case 2%, change to 0.1% and flows shifted to continue
1b. Reduce groundwater use by half	Base case proportion of population exposed to groundwater was 25%, changed to 12.5%
2a. Reduce exposure to local drain (i.e., Cover drains)	Change exposure from 35% to 5% population exposed to local drain.
3a. Toilet and ST to sewer (not drain)	Reduced toilet discharge to drain (21% to 5%) and septic tank effluent to drain (49% to 20%) to instead discharge to sewer.
3b. Improve conveyance—stop flooding and leakage	Reduced flooding from 25% to 1% and leakage from 2% to 0.1%, 99% flows continue in open drain or sewer.
3c. Improve downstream conveyance—flows to treatment	Increase flows to treatment—Sewer discharge to wastewater treatment increased from 43% to 95% and drain from 1% to 50%
3d. Improve wastewater conveyance to treatment (combine three above)	Toilet and septic tank effluent to sewer (as for 3a), improve local conveyance (3b) and discharge flows to treatment (3c).
4a. Increase sludge emptying	Increase emptying from 12% to 95%, and reduce overflow to 1%. Improved septic tank effluent $\log_{10}$ reduction due to regular emptying (see Table S2 above)
4b. Improve sludge emptying and conveyance (not treatment)	Emptying increased and improved septic tank effluent treatment (as for 4a). Reduced sludge emptied to household from 72% to 1%, increased sludge discharged to treatment from 1% to 70%.
5. Improve wastewater and faecal sludge treatment	Traditional solution—Improved both wastewater and faecal sludge to combined primary and secondary treatment (See Table S2)

6. Reduce exposure to drain and leakage, stop untreated reuse	Non-traditional solution: Reduce exposure to drain (as per 2a) reduce groundwater use (as per 1b), stop reuse of wastewater and sludge before treatment (5% from sewer, 10% drain and 1% sludge shifted from produce to treatment).

References

1. Feachem, R.; Bradley, D.; Garelick, H.; Mara, D. *Sanitation and Disease: Health Aspects of Excreta and Wastewater Management*; John Wiley & Sons: New York, NY, USA, 1983.
2. De Silva, M.; Miagostovich, M.; Victoria, M. Rotavirus and Astrovirus. In *Global Water Pathogens Project*; Rose, J.B., Jimenez-Cisneros, B., Eds.; Michigan State University: East Lansing, MI, USA.
3. Chan, M.C.; Sung, J.J.Y.; Lam, R.K.Y.; Chan, P.K.S.; Lee, N.L.S.; Lai, R.W.M.; Leung, W.K. Fecal viral load and norovirus-associated gastroenteritis. *Emerg. Infect. Dis.* **2006**, *12*, doi:10.3201/eid1208.060081.
4. Atmar, R.L.; Opekun, A.R.; Gilger, M.A.; Estes, M.K.; Crawford, S.E.; Neill, F.H.; Graham, D.Y. Norwalk virus shedding after experimental human infection. *Emerg. Infect. Dis.* **2008**, *14*, doi:10.3201/eid1410.080117.
5. Medema, G.; World Health Organization. *Risk Assessment of Cryptosporidium in Drinking Water*; World Health Organisation: Geneva, Switzerland, 2009.
6. Chappell, C.L.; Okhuysen, P.C.; Sterling, C.R.; Wang, C.; Jakubowski, W.; Dupont, H.L. Infectivity of cryptosporidium parvum in healthy adults with pre-existing anti-C. parvum serum immunoglobulin G. *Am. J. Trop. Med. Hyg.* **1999**, *60*, 157–164.
7. Platts-Mills, J.A.; Babji, S.; Bodhidatta, L.; Gratz, J.; Haque, R.; Havt, A.; McCormick, B.J.; McGrath, M.; Olortegui, M.P.; Samie, A.; et al. Pathogen-specific burdens of community diarrhoea in developing countries: A multisite birth cohort study (MAL-ED). *Lancet Glob. Health* **2015**, *3*, 564–575.
8. Pham-Duc, P.; Nguyen-Viet, H.; Hattendorf, J.; Zinsstag, J.; Phung-Dac, C.; Zurbrügg, C.; Odermatt, P. Ascaris lumbricoides and Trichuris trichiura infections associated with wastewater and human excreta use in agriculture in Vietnam. *Parasitol. Int.* **2013**, *62*, 172–180.
9. Rose, C.; Parker, A.; Jefferson B.; Cartmell, E. The characterization of feces and urine: A review of the literature to inform advanced treatment technology. *Crit. Rev. Environ. Sci. Technol.* **2015**, *45*, 1827–1879.
10. Stenström, T.A.; Seidu, R.; Ekane, N.; Zurbrügg, C. *Microbial Exposure and Health Assessments in Sanitation Technologies and Systems*; Stockholm Environment Institute (SEI): Stockholm, Sweden, 2011.
11. World Health Organization (WHO). *Guidelines for the Safe Use of Wastewater, Excreta and Greywater*; WHO: Geneva, Switzerland, 2006.
12. Von Sperling, M.; de Lemos Chernicharo, C.A. Biological wastewater treatment in warm climate regions. IWA Publishing: London, UK, 2005.
13. Jimnez, B.; Mara, D.; Carr, R.; Brissaud, F. Chapter 8: Wastewater Treatment for Pathogen Removal and Nutrient Conservation: Suitable Systems for Use in Developing Countries. In *Wastewater Irrigation and Health: Assessing and Mitigating Risk in Low-Income Countries*; Dreschel, P., Scott, A.C., Raschid-Sally, L., Redwood, M., Bahri, A., Eds.; Earthscan with the International Development Research Centre (IDRC) and the International Water Management Institute (IWMI): London, UK, 2009.
14. Verbyla, M.; von Sperling, M.; Maiga, Y. Waste Stabilization Ponds. In *Global Water Pathogens Project*; Rose, B., Jiménez-Cisneros, B., Eds; Michigan State University: East Lansing, MI, USA, 2017.
15. Cave, B.; Kolsky, P. *Groundwater Latrines and Health*; Loughborough University: Loughborough, UK, 1999.
16. Murphy, H. Persistence of Pathogens in Sewage and Other Water Types. In *Global Water Pathogens Project*; Rose, J.B., Jiménez-Cisneros, B., Eds.; Michigan State University: East Lansing, MI, USA, 2017.
17. Haas, C.; Rose, R.; Gerba, C. *Quantitative Microbial Risk Assessment*; John Wiley & Sons: New York, NY, USA, 1999.
18. Teunis, P.F.M.; Havelaar, A.H. The Beta Poisson dose-response model is not a single-hit model. *Risk Anal.* **2000**, *20*, 513–520.
19. Teunis, P.F.M.; Chappell, C.L.; Okhuysen, P.C. Cryptosporidium dose response studies: Variation between isolates. *Risk Anal*. **2002**, *22*, 175–183.
20. Teunis, P.; van den Brandhof, W.; Nauta, M.; Wagenaar, J.; van den Kerkhof, H.; van Pelt, W. A reconsideration of campylobacter dose–response relation. *Epidemiol. Infect.* **2005**, *133*, 583–592.
21. Mara, D.; Bos, R. Risk Analysis and Epidemiology: The 2006 WHO Guidelines for the Safe Use of Wastewater. In *Agriculture, Wastewater Irrigation*; International Water Management Institute: Colombo, Sri Lanka, 2010.

22. World Health Organization. *Guidelines for Drinking-Water Quality*; WHO: Geneva, Switzerland, 2011.
23. Havelaar, A.H.; Melse, J.M. *Quantifying Public Health Risk in the WHO Guidelines for Drinking-Water Quality: A Burden of Disease Approach*; Dutch National Institute for Public Health and the Environment (RIVM): Bilthoven, The Netherlands, 2003.
24. Chan, M.S. The global burden of intestinal nematode infections—Fifty years on. *Parasitol. Today* **1997**, *13*, 438–443.
25. Robb, K.; Null, C.; Teunis, P.; Yakubu, H.; Armah, G.; Moe, C.L. Assessment of fecal exposure pathways in low-income urban neighborhoods in Accra, Ghana: Rationale, design, methods, and key findings of the SaniPath study. *Am. J. Trop. Med. Hyg.* **2017**, *97*, 1020–1032.
26. Campos, L.C.; Ross, P.; Nasir, Z.A.; Taylor, H.; Parkinson, J. Development and application of a methodology to assess sanitary risks in Maputo, Mozambique. *Environ. Urban.* **2015**, *27*, 371–388.
27. Katukiza, A.Y.; Ronteltap, M.; van der Steen, P.; Foppen, J.W.A.; Lens, P.N.L. Quantification of microbial risks to human health caused by waterborne viruses and bacteria in an urban slum. *J. Appl. Microbiol.* **2014**, *116*, 447–463.
28. Fuhrimann, S.; Nauta, M.; Pham-Duc, P.; Tram, N.T.; Nguyen-Viet, H.; Utzinger, J.; Cissé, G.; Winkler, M.S. Disease burden due to gastrointestinal infections among people living along the major wastewater system in Hanoi, Vietnam. *Adv. Water Resour.* **2017**, *108*, 439–449.
29. Labite, H.; Lunani, I.; van der Steen, P.; Vairavamoorthy, K.; Drechsel, P.; Lens, P. Quantitative microbial risk analysis to evaluate health effects of interventions in the urban water system of Accra, Ghana. *J. Water Health* **2010**, *8*, 417–430.
30. World Health Organization. *Quantitative Microbial Risk Assessment: Application for Water Safety Management*; World Health Organisation: Geneva, Switzerland, 2016.
31. Westrell, T.; Schönning, C.; Stenström, T.A.; Ashbolt, N.J. QMRA (quantitative microbial risk assessment) and HACCP (hazard analysis and critical control points) for management of pathogens in wastewater and sewage sludge treatment and reuse. *Water Sci. Technol.* **2004**, *50*, 23–30.
32. Water, Engineering and Development Centre (WEDC). *SFD Report: Dhaka, Bangladesh*; WEDC: Loughborough, UK, 2016.
33. Fuhrimann, S.; Nauta, M.; Pham-Duc, P.; Tram, N.T.; Nguyen-Viet, H.; Utzinger, J.; Cissé, G.; Winkler, M.S. Disease burden due to gastrointestinal pathogens in a wastewater system in Kampala, Uganda. *Microb. Risk Anal.* **2016**, *4*, 16–28.
34. Williams, A.R.; Overbo, A. *Unsafe Return of Human Excreta to the Environment: A Literature Review*; The Water Institute at UNC: Chapel Hill, NC, USA, 2015.
35. Jimenez-Cisneros, B.E.; Maya-Rendon, C. *Communicating Current Research and Educational Topics and Trends in Applied Microbiology*; Formatex Research Centre: Badajoz, Spain, 2007.
36. Henze, M.; Harremoes, P.; Cour Jansen, J.; Arvin, E. *Wastewater Treatment: Biological and Chemical Processes*; Springer Science & Business Media: New York, NY, USA, 2001.
37. Seidu, R.; Heistad, A.; Amoah, P.; Drechsel, P.; Jenssen, P.D.; Stenström, T.A. Quantification of the health risk associated with wastewater reuse in Accra, Ghana: A contribution toward local guidelines. *J. Water Health* **2008**, *6*, 461–471.
38. Antwi-Agyei, P.; Biran, A.; Peasey, A.; Bruce, J.; Ensink, J. A faecal exposure assessment of farm workers in Accra, Ghana: A cross sectional study. *BMC Public Health* **2016**, *16*, doi:10.1186/s12889-016-3266-8.
39. Gumbo, J.; Malaka, E.M.; Odiyo, J.O.; Nare, L. The health implications of wastewater reuse in vegetable irrigation: A case study from Malamulele, South Africa. *Int. J. Environ. Health Res.* **2010**, *20*, 201–211.
40. Howard, G.; Pedley, S.; Tibatemwa, S. Quantitative microbial risk assessment to estimate health risks attributable to water supply: Can the technique be applied in developing countries with limited data? *J. Water Health* **2006**, *4*, 49–65.

© 2018 by the authors; licensee MDPI, Basel, Switzerland. This article is an open access article distributed under the terms and conditions of the Creative Commons by Attribution (CC-BY) license (http://creativecommons.org/licenses/by/4.0/).
